# Boost of solubility and supersaturation of celecoxib via synergistic interactions of methacrylic acid-ethyl acrylate copolymer (1:1) and hydroxypropyl cellulose in ternary amorphous solid dispersions

**DOI:** 10.1016/j.ijpx.2022.100115

**Published:** 2022-03-24

**Authors:** Florian Pöstges, Kevin Kayser, Edmont Stoyanov, Karl G. Wagner

**Affiliations:** a Department of Pharmaceutical Technology and Biopharmaceutics, University of Bonn, Gerhard-Domagk-Str. 3, 53121 Bonn, Germany; b Nisso Chemical Europe GmbH, Berliner Allee 42, 40212 Düsseldorf, Germany

**Keywords:** Supersaturation, Solubility enhancement, Ternary amorphous solid dispersion, Hydroxypropyl cellulose, Hot melt extrusion, Vacuum compression molding

## Abstract

A current trend in the development of amorphous solid dispersions (ASDs) is the combination of two polymers for synergistic enhancement in supersaturation of poorly soluble drugs. We investigated the supersaturation potential of celecoxib (CXB) using combinations of methacrylic acid-ethyl acrylate copolymer (1:1) (EL 100–55) and hydroxypropyl cellulose (HPC) SSL. Initially, the supersaturation potential of single polymers and combinations in various ratios was assessed. While EL 100–55 and HPC SSL alone showed limited potential in solubility enhancement of CXB the combination of both polymers led to a boost of CXB solubility, whereby most promising results were obtained using a 50:50 polymer ratio. Binary and ternary CXB ASDs (10% drug load) were prepared via vacuum compressing molding (VCM) and hot melt extrusion (HME). ASDs were studied by exploring the miscibility and intermolecular interactions and tested for their dissolution performance. HPC SSL was identified to be a suitable precipitation inhibitor when added to a fast dissolving CXB: EL 100–55 ASD. Ternary ASDs showed even further dissolution improvement, when processed by HME. The combination of heat and shear stress led to a homogeneous and intimate mixture of EL 100–55 and HPC SSL, resulting in formation of synergistic interactions with pronounced impact on CXB supersaturation.

## Highlights

• Ternary ASDs showed superior supersaturation compared to binary ASDs.
• Shear forces of HME required for synergistic polymer-polymer interactions.
• Interactions of polymers responsible for maximum solubility enhancement of celecoxib.

## Graphical abstract

**Figure:**
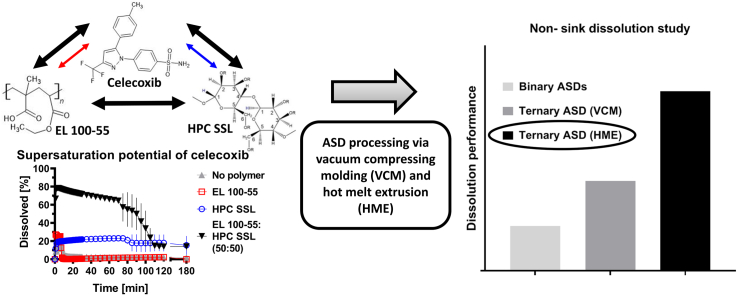

## 1 Introduction

Increasing solubility of biopharmaceutical classification system (BCS) class II drugs in combination with a stabilization of the supersaturated state in solution is widely discussed as governing mechanism for enhancing bioavailability (Alonzo et al., 2010; Brouwers et al., 2009; Hirlak et al., 2021; López Mármol et al., 2021). Amongst many enabling approaches amorphous solid dispersions (ASD) and particular molecular dispersions of a drug within a polymer matrix are an established method to enhance solubility in aqueous media (Bhujbal et al., 2021; Chiou and Riegelman, 1971). Thereby, the dissolution performance of an ASD is mostly affected by the state of supersaturation of dissolved drug (Van den Mooter, 2012; Vasconcelos et al., 2007). For an optimal performance, the polymer should be able to generate a supersaturated solution rapidly and ideally maintain the state of supersaturation over a long period of time (Guzmán et al., 2007; Sun and Lee, 2013). However, generation and maintenance of a supersaturated solution is highly dependent on specific drug-polymer interactions (Baghel et al., 2018a; Warren et al., 2010; Wilson et al., 2020). In this context, the ASD forming polymer is not necessarily the ideal polymer to stabilize supersaturation in aqueous media (Bachmaier et al., 2021; Monschke and Wagner, 2020). Wlodarski et al. detected the potential of polyvinyl alcohol (PVA) as precipitation inhibitor for the poorly soluble drug itraconazole. However, due to a lack of solubility of itraconazole in PVA in solid state, the dissolution of the corresponding ASD did not lead to supersaturation of itraconazole (Wlodarski et al., 2018). Previous investigations on ternary systems with a combination of two polymers showed superior dissolution profiles by enhancing the initial dissolution rate and the supersaturation stabilization (Baghel et al., 2018b; Borde et al., 2021; Kapote and Wagner, 2021; Van Ngo et al., 2016; Zecevic et al., 2014). Such ternary systems can be obtained by using the supersaturation generating polymer as ASD-carrier for the drug and adding a further, supersaturating stabilizing polymer to the binary ASD formulation as an external phase. Another possibility is to include the second polymer into the ASD, forming a ternary ASD (Monschke and Wagner, 2020; Xie et al., 2017). Previous investigations combined a fast dissolving ASD with externally added hydroxypropylmethylcellulose acetate succinate (HPMCAS) as precipitation inhibitor which resulted in better dissolution performance compared to the ternary ASDs (Monschke and Wagner, 2020; Müller et al., 2021; Xie and Taylor, 2016). However, a clear assignment on functionality to each polymer as either supersaturation generating or supersaturation stabilizing as a general rule seems to be questionable, as cases of synergistic effects on extent and duration of supersaturation for certain ternary ASDs have been reported. Prasad et al. prepared a ternary ASD of indomethacin using basic butylated methacrylate copolymer (Eudragit E 100) and polyvinylpyrrolidone (PVP)-K90 as polymer combination. The authors observed synergistic interactions, leading to a an increased extent of supersaturation upon dissolution in pH 1 medium for approx. 2 h compared to the respective binary formulations (Prasad et al., 2014). Hydroxypropyl cellulose (HPC) SSL has also been proven to optimize dissolution performances in ternary ASDs. Zecevic et al. demonstrated the ability of HPC SSL to tailor supersaturation in combination with HPMCAS. The use of HPC SSL led to fast dissolving of the weak base dipyridamole at low pH, followed by superior supersaturation at higher pH controlled by HPMCAS (Zecevic et al., 2014). In general, as the potential of HPC to stabilize supersaturated solutions was also shown, (Curatolo et al., 2009; Megrab et al., 1995; Yamada et al., 1999) HPC alone or in combination with an additional polymer seems to be an interesting candidate for increasing solubility mainly by stabilization of the supersaturated state of poorly soluble drugs.

The aim of this study was to investigate ternary ASDs using HPC SSL, methacrylic acid-ethyl acrylate copolymer (1:1) (EL 100–55) as alternative pH-dependent soluble polymer, and celecoxib (CXB) as BCS class II model drug, (solubility at pH 6.8: 1.5 μg/ml (Xie and Taylor, 2016)) processed by vacuum compressing molding (VCM) and hot melt extrusion (HME). Since EL 100–55 exhibits high melt viscosity (Parikh et al., 2014), it is a challenging polymer for processing via HME, thus the addition of a solid state plasticizer is required to reduce the torque during processing and to avoid a motor overload of the extruder (Monschke et al., 2021). Zecevic et al. detected the potential of HPC SSL serving as solid state plasticizer in HPMCAS ASDs due to its low molecular weight (40,000 g/mol) and low glass transition temperature (T_g_) (Zecevic et al., 2014). Therefore, in case of miscibility of EL 100–55 and HPC SSL a simplified processability during HME was assumed.

In a first step, a supersaturation assay was conducted to study the impact of EL 100–55 and HPC SSL as single polymers on the supersaturation potential of CXB. Additionally, corresponding polymer combinations with various ratios were used to investigate the effect on the supersaturation of CXB. Based on these results, polymer placebo mixtures, binary, and ternary CXB ASDs were prepared utilizing HME and VCM. X-Ray powder diffraction (XRPD) was used for verifying completely amorphous systems. The ASD formulations and the polymer placebo mixtures were investigated by differential scanning calorimetry (DSC) to examine miscibility of the components. Fourier-transform infrared spectroscopy (FT-IR) measurements were conducted to investigate polymer-polymer interactions and drug-polymer interactions. Finally, non-sink dissolution experiments were carried out to investigate the potential superiority of ternary systems compared to the binary ASDs.

## 2 Materials and methods

### 2.1 Materials

Hydroxypropyl cellulose (HPC) SSL was kindly provided by Nippon Soda Co., Ltd. (Tokyo, Japan) and methacrylic acid-ethyl acrylate copolymer (1:1) (EL 100–55) was donated by Evonik (Darmstadt, Germany). The model drug celecoxib (CXB) was purchased from Swapnroop Drugs&Pharmaceuticals (Aurangabad, India). Di‑sodium hydrogen phosphate dihydrate and sodium dihydrogen phosphate dihydrate were purchased from Th. Geyer (Renningen, Germany). Dimethyl sulfoxide (DMSO, ≥ 99.9%) was purchased from Fisher Scientific (Geel, Belgium).

### 2.2 Supersaturation assay

The supersaturation assay was carried out utilizing a miniaturized USP dissolution apparatus II (MiniDissolution apparatus) (Zecevic and Wagner, 2013) for 180 min. Temperature was set to 37 °C and the paddle speed to 75 rpm. The investigated polymers EL 100–55, HPC SSL, and mixtures of both polymers with different ratios were pre-dissolved at a total polymer concentration of 1.25 mg/ml in 0.05 M phosphate buffer at pH 6.8. CXB was dissolved completely in DMSO at a concentration of 40 mg/ml. An amount of 100 μl of the DMSO stock solution was added to 20 ml of the aqueous polymer solution to get a defined concentration of 200 μg/ml CXB. For evaluating the absolute impact of each polymer on CXB supersaturation a data set in neat buffer without polymer addition was examined. The concentrations of dissolved CXB were determined online using an 8453 UV/VIS spectrophotometer (Agilent, Waldbronn, Germany) including correction for scattering.

### 2.3 Preparation of the polymer placebo formulations and of the ASDs by VCM and HME

Table 1 presents an overview of the preparation methods for the polymer placebo formulations (P1 and P2) and for the ASDs (10% drug load) (F1 - F5). A polymer mixture of EL 100–55: HPC SSL (50:50) was either vacuum compression molded (no shear method) or hot melt extruded (including shear stress) to obtain P1 and P2, respectively. The binary ASDs of EL 100–55 + CXB (F1) and HPC SSL + CXB (F2) were prepared using VCM. For the ternary ASD F3 a physical mixture of both polymers (50:50) + CXB was processed to an ASD via VCM. To combine both preparation techniques the ternary ASD F4 was also prepared by VCM, whereby the pre-extruded placebo formulation P2 was used as ASD-carrier. F5 presents a ternary ASD that was manufactured solely by HME.

**Table 1:** Preparation methods of polymer placebo formulations (P1 and P2), of binary ASDs (F1 and F2) and of ternary ASDs (F3 - F5).

Formulation	Components [%]				Preparation method	
	CXB	EL 100–55	HPC SSL	P2	VCM	HME
P1		50	50		X	
P2		50	50			X
F1	10	90			X	
F2	10		90		X	
F3	10	45	45		X	
F4	10			90	X	
F5	10	45	45			X

VCM was conducted using a VCM tool (MeltPrep GmbH, Graz, Austria) with a 20 mm diameter disc geometry. To ensure homogenous blends every physical mixture was prepared by utilizing a MM400 ball mill (Retsch GmbH, Haan, Germany) with 30 Hz and 3 × 5 min milling cycles. Approx. 500 mg of every blend was loaded into the VCM device and heated under vacuum for 15 min at a temperature of 160 °C. Annealing temperature was selected based on the CXB melting point of 160.9 °C (Bochmann et al., 2018). The resulting discs were milled utilizing the MM400 ball mill (Retsch GmbH, Haan, Germany) at 30 Hz and passed through a 355 μm sieve to remove larger particle fractions. To decrease milling stress on the ASDs milling time was kept to a minimum of 30 s.

**Fig. 1:**
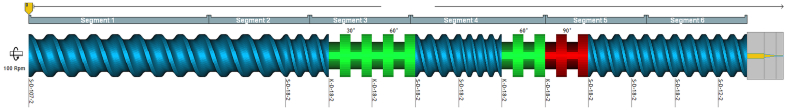
Screw configuration of the extruder with conveying elements (9,12, and 18 mm pitch) (blue) and kneading elements (30°, 60° (green) and 90° (red) staggering angle): Segment 1 (ambient temperature), segment 2 (40 °C), segment 3 (75 °C), segment 4, 5 and 6 (150 °C). (For interpretation of the references to colour in this figure legend, the reader is referred to the web version of this article.)

HME was conducted using a 12 mm co-rotating twin screw extruder ZE 12 (Three-Tec GmbH, Seon, Switzerland) with a functional length of 25:1 L/D and five heating zones, equipped with a 2 mm die and a fixed screw configuration, which is presented in Fig. 1. Prior to the extrusion processes, physical mixtures were blended by a Turbula® mixer (Willy A. Bachofen AG Maschinenfabrik, Switzerland), spinning at 50 rpm for 10 min. Compared to VCM the maximum processing temperature of the extrusion process was reduced, as additional heat was assumed to be generated by viscous dissipation (shear forces). Therefore, the selected temperatures for the five heating zones were 40/75/150/150/150 °C. The blends were fed to the twin screw extruder at a constant rate of 2 g/min and processed at a constant screw speed of 100 rpm.

The obtained extrudates were milled utilizing a MM400 ball mill (Retsch GmbH, Haan, Germany) at 30 Hz for 30 s and passed through a 355 μm sieve to remove larger particles.

### 2.4 X-ray powder diffraction (XRPD)

Neat CXB and the milled ASDs were examined in reflection mode and in a 4–45° 2θ range with a step size of 0.017° 2θ using an X'Pert MRD Pro (PANalytical, Almelo, Netherlands). Experiments were performed with an X'Celerator detector and nickel filtered CuKα1 radiation at 45 kV and 40 mA.

### 2.5 Differential scanning calorimetry (DSC)

DSC analysis of the polymers, polymer placebo formulations (P1 and P2), and ASDs (F1 - F5) were performed by using a DSC 2 instrument (Mettler, Gießen, Germany), equipped with nitrogen cooling system. Approx. 10 mg of the samples were weighted into an aluminum pan with a pierced lid. Analysis was conducted in TOPEM- mode, a temperature- modulated program, with a constant temperature rising of 2 °C/ min from 0 °C to 170 °C. All experiments were carried out in triplicates.

### 2.6 Fourier-transform infrared spectroscopy (FT-IR)

Solid state molecular interactions were examined using a Spectrum Two FT-IR spectrometer (PerkinElmer, Waltham, MA, USA). Raw materials, physical mixtures, polymer placebo formulations (P1 and P2), and ASDs (F1- F5) were analyzed in a spectral range of 450–4000 cm^−1^.

### 2.7 Non-sink dissolution study

Non-sink dissolution experiments were carried out by using the MiniDissolution apparatus (Zecevic and Wagner, 2013) with 20 ml 0.05 M phosphate buffer (pH 6.8) at 37 °C and a paddle speed of 75 rpm for 180 min. A sample size of 40 mg ASD was used for dissolution experiments to obtain a target CXB content of 4 mg (theoretical concentration of 200 μg/ml) in each vessel. Concentrations of dissolved CXB were determined online using an 8453 UV/VIS spectrophotometer (Agilent, Waldbronn, Germany) including correction for scattering.

## 3 Results

### 3.1 Supersaturation assay

**Fig. 2:**
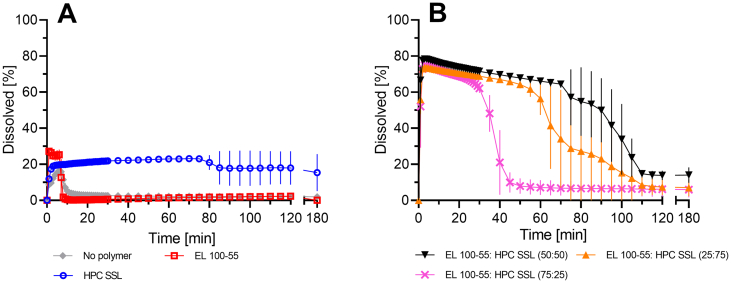
Supersaturation assay of 0.2 mg/ml CXB (= 100%) in 20 ml 0.05 M phosphate buffer at pH 6.8 (37 °C, 75 rpm paddle speed): (A) dependent on pre-dissolved single polymers  EL 100–55,  HPC SSL, and  without polymer; (B) dependent on pre-dissolved EL 100–55: HPC SSL mixtures in various ratios ▼ 50:50,  75:25, and  25:75. For each experiment (*n* = 3), the total polymer concentration was 1.25 mg/ml.

Fig. 2 represents the supersaturation assay of neat CXB, in presence of pre-dissolved single polymers (Fig. 2A), and in presence of polymer mixtures, using various ratios of polymers (25:50, 50:50, 75:25) (Fig. 2B). Without any pre-dissolved polymer, a maximum CXB concentration of 16% was measured after 6 min, followed by a sudden decrease to less than 1%. By pre-dissolving EL 100–55 the supersaturation behavior of CXB was only slightly enhanced, since the initial concentration of about 25% CXB plummeted to almost 0% after 7 min. A comparable extent of supersaturation (approx. 20%) was achieved by using HPC SSL. However, the supersaturated state was stabilized over the entire observation period of 180 min with only a slight decline of CXB concentration to 18% at the end of the test.

A combination of EL 100–55: HPC SSL (50:50) showed an initial boost of solubility to almost 80%. That extent was maintained for up to 70 min with only a slight decrease in concentration, followed by a more pronounced reduction of concentration to approx. 15% after 110 min. In order to evaluate the supersaturation potential for different relative ratios of EL 100–55 and HPC SSL two further polymer ratios (25:75 and 75:25) were assessed. The initial boost of supersaturation was only slightly affected by changes in the ratio of the two polymers. Both a relative excess of EL 100–55 and a relative excess of HPC SSL led to an initial solubility between 75% and 80% CXB. However, differences were observed in terms of maintaining the supersaturation state. By pre-dissolving EL 100–55: HPC SSL in a ratio of 75:25, the supersaturation was only stabilized for about 30 min. The polymer mixture EL 100–55: HPC SSL in a ratio of 25:75 maintained the supersaturated state for about 55 min and therefore, showed also lower potential in stabilizing the supersaturated state of CXB compared to the polymer mixture in a ratio of 50:50. Since the balanced mass ratio of the polymers showed the most stable supersaturation, the polymer placebo mixtures (P1 and P2) and the ternary ASDs (F3- F5) were prepared with a polymer ratio of 50:50.

### 3.2 X-ray powder diffraction (XRPD)

**Fig. 3:**
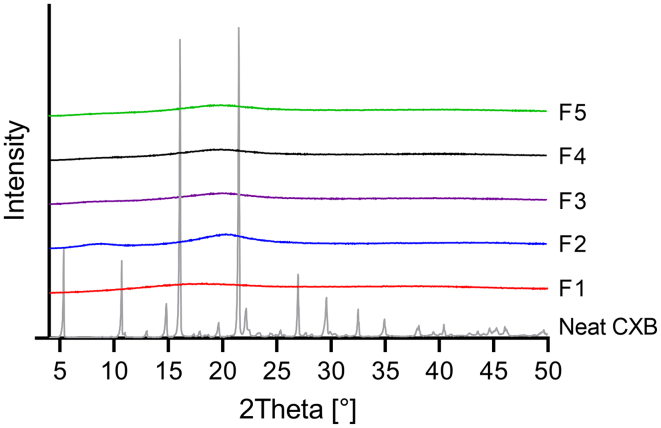
X-ray powder diffraction (XRPD) diffractograms of neat CXB and ASDs: EL 100–55 (F1), HPC SSL (F2), EL 100–55: HPC SSL VCM (F3), EL 100–55: HPC SSL HME polymer extrudate VCM (F4) and EL 100–55: HPC SSL HME (F5).

Fig. 3 represents the X-ray powder diffraction (XRPD) diffractograms of neat CXB and formulated ASDs. The crystalline structure of neat CXB was confirmed by the sharp reflection peaks. The observed absence of reflection peaks in all ASD samples indicated the complete amorphous character of processed CXB.

### 3.3 Differential scanning calorimetry (DSC)

**Fig. 4:**
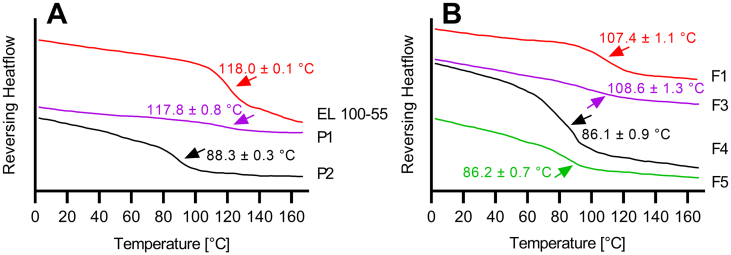
A: Differential scanning calorimetry (DSC) thermograms (exo up) of EL 100–55, polymer placebo melt (P1), and polymer placebo extrudate (P2). B: DSC thermograms of CXB ASDs of EL 100–55 (F1), EL 100–55: HPC SSL VCM (F3), EL 100–55: HPC SSL HME polymer extrudate VCM (F4) and EL 100–55: HPC SSL HME (F5).

To evaluate miscibility of EL 100–55 and HPC SSL, glass transition temperatures (T_g_) of pure polymers and of the polymer placebo formulations P1 and P2 were investigated (Fig. 4A). EL 100–55 exhibited a T_g_ at 118.0 ± 0.1 °C. Unfortunately, our DSC method was not able to detect a T_g_ for HPC SSL, which is a well-known issue of HPC. Therefore, we used a T_g_ for HPC SSL which was recently published by Luebbert et al. as a predicted T_g_ of 81.8 °C (Luebbert et al., 2020). Our DSC thermogram of HPC SSL can be found in the Supplementary Data (Fig. S1A). The polymer placebo melt P1 processed by VCM showed a glass transition at 117.8 ± 0.8 °C, very close to the T_g_ of EL 100–55, indicating the inability of forming a single phased system. However, the placebo formulation P2 processed by HME showed a single glass transition at 88.3 ± 0.3 °C, indicating miscibility of the polymers and formation of a single phase under the influence of heat and shear.

The DSC thermograms of the ASDs are given in Fig. 4B. The DSC measurements of the CXB: EL 100–55 ASD (F1) showed a single T_g_ at 107.4 ± 1.1 °C, indicating the formation of a single phased ASD. Due to the above-mentioned problem of determining the T_g_ of HPC SSL, the T_g_ of the CXB: HPC SSL ASD (F2) could not be determined. The corresponding DSC thermogram of F2 can be found in the Supplementary Data (Fig. S1B). The vacuum compression molded ternary ASD (F3) exhibited a T_g_ at 108.6 ± 1.3 °C. Similar to the investigations of the polymer placebo formulations, VCM did not lead to a single phased system, since a similar T_g_ was detected in the CXB: EL 100–55 ASD (F1). The pre-extruded ternary VCM ASD (F4) showed a single glass transition at 86.1 ± 0.9 °C, indicating the formation of a single phased system. Similar results were obtained for the hot melt extruded ternary ASD (F5) with a T_g_ of 86.2 ± 0.7 °C.

### 3.4 Fourier-transform infrared spectroscopy (FT-IR)

**Fig. 5:**
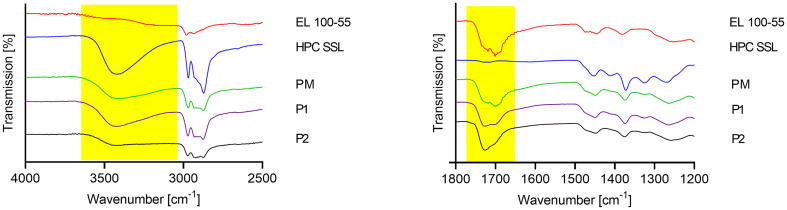
Fourier-transform infrared (FT-IR) spectra of pure polymers, physical polymer mixture (PM), polymer placebo melt (P1), and polymer placebo extrudate (P2).

The FT-IR measurement results of the polymers, the physical polymer mixture (50:50), and the polymer placebo formulations P1 and P2 are given in Fig. 5. For better visualization of the FT-IR results the spectra are presented in two graphs, the left presenting the wavenumbers from 2500 to 4000 cm^−1^, and the right the wavenumbers from 1200 to 1800 cm^−1^. No peaks were visible between 1800 cm^−1^ and 2500 cm^−1^.

For EL 100–55 the CH stretching was represented by the peak at 2930 cm^−1^. The double peak at about 1701 cm^−1^ and 1719 cm^−1^ corresponded to the C=O stretching vibrations of the carboxylic acid groups and to the esterified carboxyl groups, respectively, whereby the band at 1701 cm^−1^ was more intensive. The peaks between 1350 cm^−1^ and 1500 cm^−1^ were attributed to CH vibrations.

For HPC SSL a broad OH stretching at 3430 cm^−1^ showed OH vibrations. OCHX and CH vibrations were observed at 2970 cm^−1^.

Regarding the physical mixture of both polymers, the characteristic peaks of each polymer were identified again. The OH peak of HPC SSL and the C=O double peak of EL 100–55 were clearly detected in the FT-IR spectrum of the mixture. Regarding P1, the broad OH peak did not change in shape or intensity. However, the C=O double peak changed slightly, whereby the peak at 1726 cm^−1^ intensified compared to the one at 1701 cm^−1^. These changes within the FT-IR spectrum of P1 indicated specific interactions between EL 100–55 and HPC SSL when processed by VCM. The carbonyl moiety of EL 100–55 interacted with HPC SSL, whereby no indication for participation of the hydroxy groups of HPC SSL was given. However, changes in the FT-IR spectrum of P2 were clearly more pronounced. The intensity of the OH band of HPC SSL was strongly decreased and the shape of the C=O double peak changed even more compared to the FT-IR spectrum of P1, as the peak intensity at 1701 cm^−1^ was even more reduced. These observations indicated strong interactions between EL 100–55 and HPC SSL, when processed by HME, whereby the hydroxy groups of HPC SSL were clearly involved and the C=O of EL 100–55 participated stronger than in P1.

**Fig. 6:**
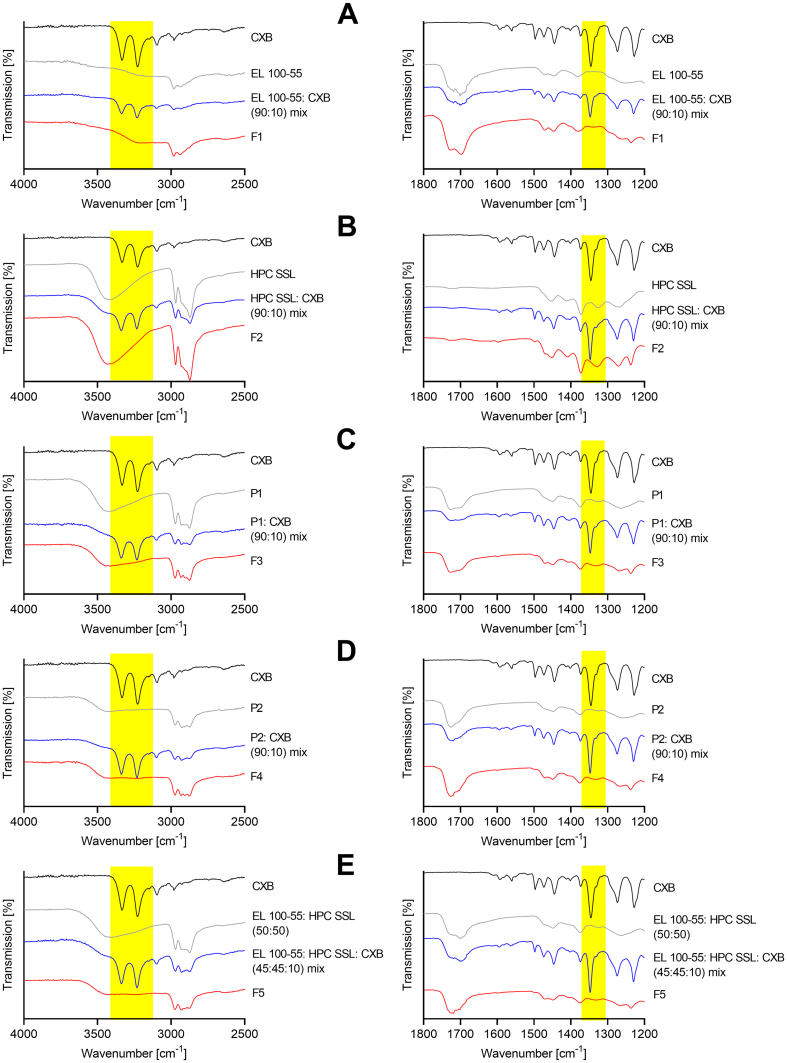
Fourier-transform infrared (FT-IR) spectra of the CXB formulations: (A) EL 100–55 (F1), (B) HPC SSL (F2), (C) EL 100–55: HPC SSL VCM (F3), (D) EL 100–55: HPC SSL HME polymer extrudate VCM (F4) and (E) EL 100–55: HPC SSL HME (F5). ASDs are compared to neat CXB, the corresponding ASD forming polymer(s) and the ASD forming polymer(s) + CXB.

In Fig. 6 the FT-IR spectra of CXB, of the ASD forming polymer(s), of the ASD forming polymer(s) + CXB, and of the ASDs (F1 - F5) are given. The FT-IR measurement of CXB showed NH stretching vibrations at 3333 cm^−1^ and 3227 cm^−1^ and S=O stretching at 1346 cm^−1^. The peak at 1228 cm^−1^ indicated a CF stretching. In all investigated ASDs the NH bands and S=O bands of CXB completely vanished, indicating the presence of molecular interactions between the polymers and CXB under participation of the sulfonamide structure of CXB. Regarding F1 (Fig. 6A) the C=O double peak of EL 100–55 shifted only slightly in shape (from 1701 to 1698 cm^−1^ and from 1719 to 1727 cm^−1^), whereby the intensities in the double peak did not change compared to each other. This observation indicated interactions between EL 100–55 and CXB with only negligible participation of the carbonyl moiety of EL 100–55. For F2 (Fig. 6B) no shift or shape change was detected for the OH band, indicating that the hydroxy group was not involved in specific interactions with CXB. Regarding F3 (Fig. 6C), the broad OH band at 3430 cm^−1^ was still visible and C=O double peak did not change compared to P1. Therefore, interactions between the polymers and CXB with participation of hydroxy and carbonyl moiety of HPC SSL and EL 100–55, respectively, were not observed. Similar results were obtained for the extruded ternary ASDs F4 and F5. The FT-IR spectra of CXB containing F4 (Fig. 6D) and F5 (Fig. 6E) showed no further interactions compared to the FT-IR spectrum of pure polymer extrudate P2, as interactions between the polymers and CXB with participation of hydroxy and carbonyl moiety of extruded polymers were not observed.

### 3.5 Non-sink dissolution study

**Fig. 7:**
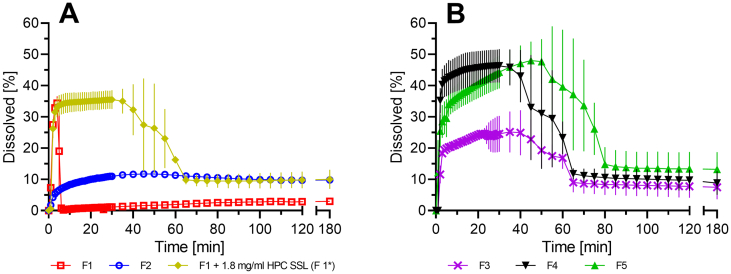
Dissolution profiles of binary CXB ASDs (A) of  EL 100–55 (F1),  HPC SSL (F2),  EL 100–55 in presence of 1.8 mg/ml pre-dissolved HPC SSL (F1*) and ternary CXB ASDs (B) of  EL 100–55: HPC SSL VCM (F3), ▼ EL 100–55: HPC SSL HME polymer extrudate VCM (F4),  EL 100–55: HPC SSL HME (F5). Non-sink dissolution study was conducted in 20 ml 0.05 M phosphate buffer at pH 6.8 (37 °C, 75 rpm paddle speed).

Fig. 7 represents the non-sink dissolution study of binary CXB ASDs (Fig. 7A) and ternary CXB ASDs (Fig. 7B). The CXB: EL 100–55 ASD (F1) showed a fast dissolution rate, as 34% of dissolved CXB was detected after 4 min. However, the concentration plummeted directly to less than 2%, demonstrating that EL 100–55 alone was not capable of keeping CXB in solution. The HPC SSL ASD (F2) showed slower release of CXB and a rather low maximum solubility of 12%, but the capability of maintaining the supersaturated state. Based on these observations, it was decided to further investigate the dissolution behavior of the fast-dissolving CXB: EL 100–55 ASD in presence of 1.8 mg/ml pre-dissolved HPC SSL (F1*). The amount of pre-dissolved HPC SSL corresponded to the amount of EL 100–55 that was present within the CXB: EL 100–55 ASD. The first minutes of the dissolution were comparable to F1, since the fast dissolution rate was observed again. However, the pre-dissolved HPC SSL prevented the early precipitation and prolonged the supersaturated state up to 10 times. After 40 min precipitation started and a decrease to the concentration level of F2 was observed after 65 min. Compared to the dissolution of the CXB: EL 100–55 ASD with pre-dissolved polymer (F1*) the dissolution of the ternary ASD that was prepared solely by VCM (F3) showed a pronounced reduction of the initial CXB solubility. Although F3 showed fast dissolution rate, only 20% CXB was dissolved after 5 min. Concentration of dissolved CXB was enhanced to 25% after 40 min, followed by a reduction to 9% after 65 min. However, the ternary ASD with the pre-extruded polymer mixture (F4) showed even further improved dissolution and supersaturation performance than F1*. While the dissolution rate of F4 was faster, resulting in a higher level of dissolved CXB of about 45%, the dissolution of the ternary ASD that was solely prepared by HME (F5) showed slower release of CXB. Nevertheless, after about 35 min the same extent of supersaturation was obtained and maintained for an even longer period. After 50 min precipitation started until the concentration reached a level of 10% after 80 min.

## 4 Discussion

In this study binary and ternary ASDs of CXB, using EL 100–55 and HPC SSL as ASD-carrier, were successfully processed via VCM and/or HME, confirmed by XRPD by absents of any Bragg-diffraction peaks of CXB.

As EL 100–55 exhibits a high melt viscosity (Monschke et al., 2021; Parikh et al., 2014), it is a challenging polymer for processing through HME. However, we confirmed our assumption of using HPC SSL as solid state plasticizer, as extrusion of the polymer extrudate P2, and of the ternary ASD F5 was conducted without using an additional plasticizer.

By dissolving the binary CXB: EL 100–55 and CXB: HPC SSL ASDs, strengths and limitations of the individual polymers were revealed. EL 100–55 was able to generate a supersaturated CXB solution of 34%, but showed incapability of maintaining the supersaturated state. In contrast, HPC SSL showed lower potential in increasing the CXB solubility, however the ability to stabilize the supersaturated state.

By combining both polymers solubility and supersaturation were strongly improved due to synergistic interactions between EL 100–55 and HPC SSL in liquid state, as demonstrated by the supersaturation assay. The dissolution study identified pre-dissolved HPC SSL as a promising precipitation inhibitor for the fast dissolving binary CXB: EL 100–55 ASD (F1). HPC SSL was successfully used as a functional external additive for F1 leading to enhanced dissolution performance in terms of maintaining the supersaturation state. However, best performing ternary ASDs were only obtained when produced by HME (F4 and F5), combining heat and shear stress. The latter was decisive for the formation of a homogeneous and intimate mixture of the two polymers, as demonstrated in the DSC and FT-IR investigations of the polymer placebo mixtures (P1 and P2) and ternary ASDs (F3 - F5). While VCM did not lead to complete miscibility of EL 100–55 and HPC SSL in the polymer placebo melt (P1), the polymer placebo extrudate (P2) processed by HME showed the formation of a single phased system and strong interactions between the polymers. Compared to the extrusion process, no shear forces were applied on the molding mass during VCM. Since EL 100–55 exhibits very high melt viscosity, shear forces were apparently necessary for the formation of a single phased system. These findings were in line with the investigations of the ternary ASDs. In contrast to the vacuum compression molded ASD F3, the hot melt extruded ASDs F4 and F5 became single phased ternary ASDs. Also, stronger interactions were identified between EL 100–55 and HPC SSL when processed by HME (F4 and F5) than by VCM (F3). In this context it must be noted that VCM was a useful method for the preparation of a single phased ternary ASD, when the polymers were pre-extruded by HME (F4). Apparently, shear forces via HME were especially important for the formation of pronounced polymer-polymer interactions. Further ASD processing with CXB via VCM without shear led to a formulation (F4) that showed similar results in both the solid state analysis (DSC and FT-IR) and in the dissolution study compared to F5 that was solely manufactured by HME. Accordingly, the formation of a homogeneous and intimate mixture of EL 100–55 and HPC SSL, accompanied by strong interactions between the polymers in the solid phase, was crucial for optimal extent and maintenance of CXB supersaturation. Although many studies have emphasized the importance of drug-polymer interactions in the solid state of an ASD for dissolution enhancement (Nair et al., 2020; Prasad et al., 2014; Shi et al., 2019) from our point of view specific polymer-polymer interactions in case of ternary systems were also considered as important in terms of solubility enhancement of poorly soluble drugs.

## 5 Conclusion

Although HPC SSL alone showed only limited capability of generating supersaturated CXB solutions in aqueous media, it was highly effective in maintaining the supersaturated state in combination with EL 100–55. Since a balanced mass ratio of both polymers demonstrated the highest potential in maintaining the supersaturated state of CXB, ternary ASDs were prepared using a polymer ratio of 50:50. While neither EL 100–55 nor HPC SSL alone with CXB in binary ASDs showed promising performance in generating a high kinetic solubility or maintaining supersaturation, respectively, the synergistic interactions of the ternary ASDs resulted in a remarkable boost in solubility and supersaturation of CXB. The formation of these synergistic interactions required a homogeneous and intimate mixture of EL 100–55 and HPC SSL which required next to heat also the shear stress of HME. Best results in terms of supersaturation of CXB were achieved when all three components were processed into an ASD via HME.

## CRediT authorship contribution statement

**Florian Pöstges:** Conceptualization, Methodology, Investigation, Data curation, Writing – original draft, Writing – review & editing, Visualization. **Kevin Kayser:** Methodology, Investigation, Writing – review & editing. **Edmont Stoyanov:** Conceptualization, Resources, Writing – review & editing. **Karl G. Wagner:** Conceptualization, Resources, Writing – review & editing, Supervision.

## Declaration of Competing Interest

This work was funded and supported by Nisso Chemical Europe GmbH, Düsseldorf, Germany. University of Bonn and Nisso Chemical Europe GmbH participated in study design, research, interpretation of data, writing, data collection, analysis, reviewing, and approving the publication. Edmont Stoyanov is an employee of Nisso Chemical Europe GmbH and does not own stocks on Nippon Soda Ltd. Florian Pöstges and Kevin Kayser are PhD students and Karl G. Wagner a professor at the department of Pharmaceutical Technology and Biopharmaceutics at the University of Bonn, Germany. They have no additional conflicts of interest to report.

## Appendix A Supplementary data

Supplementary material: Differential scanning calorimetry (DSC) thermograms (exo up) of HPC SSL and CXB: HPC SSL ASD Image 11
