# Supplementary material for: Boost of solubility and supersaturation of celecoxib via synergistic interactions of methacrylic acid-ethyl acrylate copolymer (1:1) and hydroxypropyl cellulose in ternary amorphous solid dispersions
Source: Int J Pharm X. 2022 Mar 24;4:100115. doi: 10.1016/j.ijpx.2022.100115 (PMC8968008; doi:10.1016/j.ijpx.2022.100115)
Supplement: Supplementary file 1 — Supplementary material: Differential scanning calorimetry (DSC) thermograms (exo up) of HPC SSL and CXB: HPC SSL ASD [file mmc1.pdf]

Supplementary Materials:

Boost of solubility and supersaturation of celecoxib via synergistic interactions of methacrylic acid-ethyl acrylate copolymer (1:1) and hydroxypropyl cellulose in ternary amorphous solid dispersions

Florian Pöstges [a], Kevin Kayser [a], Edmont Stoyanov [b], Karl G. Wagner [a, *]

[a] *Department of Pharmaceutical Technology and Biopharmaceutics, University of Bonn, Gerhard-Domagk-Str. 3, 53121 Bonn, Germany*

[b] *Nisso Chemical Europe GmbH, Berliner Allee 42, 40212 Düsseldorf, Germany*

* Corresponding author: karl.wagner@uni-bonn.de (Karl G. Wagner)

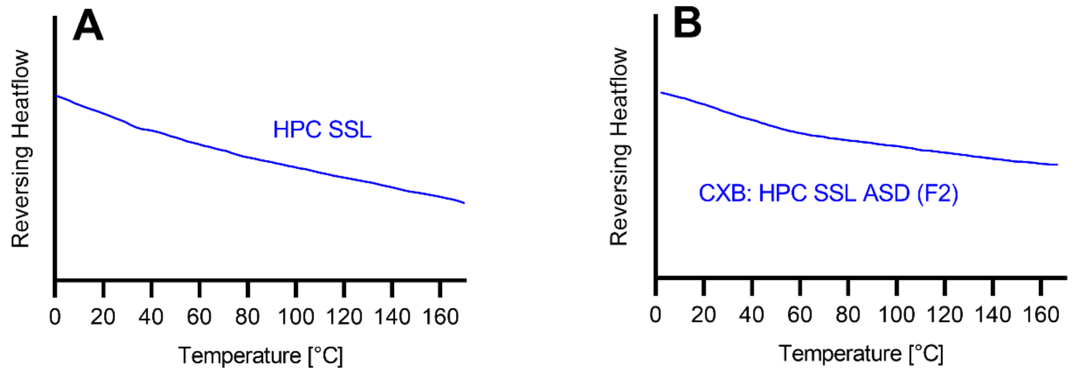

Figure S1: Differential scanning calorimetry (DSC) thermograms (exo up) of **(A)** HPC SSL and of **(B)** CXB: HPC SSL ASD (F2)
